# Soil aggregates regulate microbial drivers of phosphorus fractions under mowing and phosphorus addition

**DOI:** 10.3389/fmicb.2025.1671636

**Published:** 2025-10-30

**Authors:** Xiuping Li, Haiying Cui, Shanling Wang, Baoshuang Hu, Huiliang Zhai, Jiaxin Hu, Xia Peng, Muhammad Shakir, Wei Sun

**Affiliations:** Key Laboratory of Vegetation Ecology, Institute of Grassland Science, School of Life Science, Northeast Normal University, Ministry of Education, Jilin Songnen Grassland Ecosystem National Observation and Research Station, Changchun, China

**Keywords:** P cycling, soil structure, soil microorganisms, land management practices, grassland

## Abstract

Microorganisms play a vital role in maintaining ecosystem structure and function by mediating the dynamics of phosphorus (P) fractions under land-use intensification. However, microbial properties vary considerably across different soil aggregate sizes, making it challenging to determine how microorganisms regulate P fractions in response to mowing and P addition. To address this knowledge gap, we conducted an 8-year field study in a meadow steppe in Northeast China to examine the effects of mowing, P addition, and their interaction on P fractions across soil aggregate sizes. The results indicated that the interaction between mowing and P addition increased total P across soil aggregates. Specifically, this interaction enhanced available P (the sum of labile Pi and labile Po) by 74.13, 55.01, and 9.50% in large macroaggregates (LMA), small macroaggregates (SMA), and microaggregates (MA), respectively. In LMA, increases in moderately labile Pi, moderately labile Po, and residual P were driven by a decreased microbial biomass carbon to microbial biomass phosphorus (MBC: MBP) ratio, which was reduced by P addition. In SMA, P addition and mowing increased labile Po, labile Pi, and moderately labile Pi by enhancing plant richness. In MA, P addition not only increased labile Po and moderately labile Pi by stimulating plant belowground biomass (BGB) but also enhanced residual P through elevated alkaline phosphatase (ALP) activity. These findings highlight the critical role of soil aggregates in regulating the dynamics of microbially driven soil P fractions. Overall, the distinct responses of P fractions and their dominant drivers provide valuable insights into P cycling in grasslands and support the development of sustainable land management strategies.

## Introduction

Phosphorus (P) is an essential nutrient that supports and sustains ecosystem productivity (Yang et al., 2021). However, its limitation is widespread across terrestrial ecosystems globally, with approximately 37% of grasslands being P-limited (Hou et al., 2020). Therefore, understanding the mechanisms that control soil P bioavailability is crucial for alleviating global terrestrial P limitations. In soils, phosphate anions in the soil solution can adsorb onto the positively charged surfaces of iron (Fe) and aluminum (Al) oxides/hydroxides, as well as kaolinite minerals, resulting in the formation of moderately stabilized P fractions (Lambers et al., 2015). Alternatively, phosphate anions can react with calcium ions (Ca^2+^) to precipitate, forming more stable P fractions (Cao and Harris, 2008). These insoluble P fractions, which vary in solubility, serve as reservoirs of available P through their gradual and controlled release (Helfenstein et al., 2018).

Soil microorganisms play a crucial role in driving the release of insoluble P fractions and enhancing soil P availability (Rawat et al., 2021; Richardson and Simpson, 2011). Soil microbial biomass phosphorus (MBP) is a key component of total soil P and generally equals or exceeds the P concentration in plant biomass (Turner et al., 2013). The P released from microorganisms’ necromass significantly affects the levels of soil P fractions (Chen et al., 2023). Radiotracer experiments have demonstrated that the addition of P fertilizers enhances soil MBP levels, thereby increasing soil P bioavailability (Spohn and Kuzyakov, 2013; Spohn and Widdig, 2017). Conversely, plant biomass removal shows a negative effect on soil MBP (Bie et al., 2024). Alkaline phosphatase (ALP) is an extracellular enzyme synthesized and secreted by soil microorganisms, regulating soil P fractions by mineralizing organic P (Li et al., 2021). ALP activity is lower in natural grasslands than in agroecosystems that use fertilizers (Lu et al., 2022). In addition to ALP activity, which decreases as soil organic carbon declines, the removal of plant biomass further reduces soil nutrients (Katsalirou et al., 2016). Most research on soil P cycling has predominantly concentrated on bulk soil. However, microbial properties vary significantly across different soil aggregates.

Soil aggregates shape the microbial drivers of P fractions, leading to distinct microbial regulation of soil P dynamics across aggregate sizes (Wilpiszeski et al., 2019). As the size of aggregates decreases, bacterial abundance and extracellular enzyme activities increase, suggesting that microaggregates (MAs) may serve as the primary habitat for microorganisms (Tian et al., 2022). However, studies have shown that the majority (90%) of soil bacteria reside in large macroaggregates (LMAs) (Ranjard et al., 2000). Mowing impeded the formation of large macroaggregates (LMAs) owing to the decline in soil organic carbon caused by plant biomass removal (Wen et al., 2016). Reserves of labile Pi and total P in soil were significantly depleted after long-term mowing treatments (Boitt et al., 2018). P fertilization did not affect soil aggregate formation but increased labile Pi and Po, moderately labile Pi and Po, and residual and total P in all aggregate sizes (Khan et al., 2023). Mowing and P addition can directly influence microbial properties by altering the proportion of soil aggregates. However, the understanding of how these factors (mowing and phosphorus) affect soil aggregates and their associated microbial properties, along with subsequent microbial regulation of P fractions, remains limited.

The Songnen Grassland in northern China is a vital part of the Eurasian steppe, providing various ecological functions, with forage grass provision being one of its primary roles. However, the low availability of soil P, largely due to soil salinization in the region, limits both hay production and quality. Therefore, we monitored soil P fractions across various soil aggregate sizes following long-term (eight-year) mowing and P addition treatments. The primary objective was to investigate the effects of mowing and P addition on the P fractions and their drivers in different soil aggregate sizes. We hypothesized that (I) phosphorus (P) fertilization would increase P fractions and total P, whereas mowing would decrease total P by removing P in plant biomass, and (II) the drivers of P fractions would vary across different soil aggregates.

## Materials and methods

### Study site and experimental design

This study was conducted in a semi-arid meadow steppe in northeastern China (48°05′N, −126°20′E), which experiences a continental monsoon climate. The rainy season is predominantly concentrated in July and August. The mean annual temperature is 6.6°C, and the average precipitation is 441.0 mm/year over the period from 2020 to 2022, sourced from the China Meteorological Information Center.^1^ The soil is covered by a white clay layer, and the pH is ≥ 8.5, which is classified as Solonetz based on the FAO classification. The majority of plants here are species adapted to solonetz soil, such as *Leymus chinensis, Suaeda glauca, Phragmites australis, and Melilotus officinalis.*

In 2015, a 2-hectare study field was established using a randomized block design. A total of six 35 m × 35 m blocks were established, each divided into two plots (10 m × 5 m) corresponding to control and P addition. Each plot was further divided into two subplots (5 m × 5 m), representing mown and unmown treatments. Overall, each block included four treatments: control, mowing, P addition, and their interaction (P addition × mowing). For each treatment, six replicates were included, and samples were collected from a total of 24 subplots. Edge effects were minimized by implementing 10-meter grass buffers around blocks and 1-meter buffers around plots. P fertilizer was applied at a rate of 5 g m^−2^ yr.^−1^, in line with atmospheric P deposition rates in northern China (Elser et al., 2007; He et al., 2007). From May to September, calcium P (Ca_3_(PO_4_)_2_) fertilizer was applied monthly in five equal portions via water-soluble spraying, while the control plot was sprayed with water of the same volume. Mowing was performed once annually in August.

### Plant and soil sampling

In late July 2022, aboveground plant biomass (AGB) was sampled from a random (0.5 m × 0.5 m) area in each subplot. Belowground biomass (BGB) was sampled using a sampling device measuring 50 cm in length and 12 cm in width, inserted to a depth of 30 cm, and subsequently washed to remove soil matrices. Both AGB and BGB were oven-dried at 65°C for 72 h and weighed. Plant species richness was evaluated by surveying three permanent vegetation survey quadrats (0.5 m × 0.5 m) established in 2015 in each subplot, with the mean value representing the species richness of the plot. During plant sampling, five random soil samples (3 cm diameter × 10 cm depth) were collected and homogenized into a composite soil sample. According to the dry sieving method (Jiang et al., 2013; Bach and Hofmockel, 2014), the soil sample was separated into LMA (>2000 μm), small macroaggregates (SMA) (250–2000 μm), and MA (<250 μm) using a sieve analyzer (AS200, Shanghai, China). Each aggregate fraction was divided into two parts: one part was air-dried, and the other part was stored at −80°C to maintain freshness.

### Soil aggregates: physical and chemical properties

Soil aggregate water content (SWC) was determined by the weight loss between the fresh sample (~15.0 g) and its dry weight after oven-drying at 105°C for 24 h. For soil aggregate pH, the sample was homogenized with 50 mL of deionized water (ratio: 1:5 sample to deionized water) and measured using a pH electrode (PHS-3E, Shanghai, China). To measure soil aggregate available N concentration, the sample was extracted with 2 M potassium chloride (KCl) solution and analyzed using an automated discontinuous flow analyzer (Futura II, Guangzhou, China).

### Soil P fractions measurement at aggregate levels

Soil P fraction concentrations were measured using the Hedley sequential extraction method (Hedley et al., 1982). Briefly, 0.500 g of air-dried soil aggregate was sequentially extracted with an anion exchange resin bag (0.400 g), 0.5 M sodium bicarbonate (NaHCO_3,_ pH 8.5), 0.1 M sodium hydroxide (NaOH), 0.1 M NaOH (secondary extraction), and 1 M hydrochloric acid (HCl), followed by digestion at 370°C for 1 h. Then, the extraction solutions were analyzed using an automated discontinuous flow analyzer (SmartChem 450, AMS, Italy) to quantify resin phosphorus (Resin-P), NaHCO_3_ inorganic phosphorus (NaHCO₃ Pi), NaOH inorganic phosphorus (NaOH Pi), NaOHus secondary inorganic phosphorus (NaOHus Pi), HCl inorganic phosphorus (HCl Pi), and residual phosphorus (residual-P). Each extraction solution was further digested with 0.5 g potassium persulfate (K_2_H_2_O_8_) at 120°C for 1 h to determine total phosphorus (Pt) in the NaHCO_3_, NaOH, NaOHus, and HCl extracts. Organic phosphorus (Po) in each extract—NaHCO_3_ Po, NaOH Po, NaOHus Po, and HCl Po—was then calculated as the difference between total Pt and Pi.

We further defined these soil P fractions according to their characteristics and bioavailability. Resin-P and NaHCO_3_-Pi were combined and defined as labile Pi. NaHCO_3_-Po was defined as labile Po. The sum of NaOH-Pi, NaOHus-Pi, and HCl-Pi was classified as moderately labile Pi, while the sum of NaOH-Po, NaOHus-Po, and HCl-Po was classified as moderately labile Po. Residual P represents the fraction that is least available to organisms in the ecosystem (Fan et al., 2019; García‐Velázquez et al., 2020). Pt, Pi, and Po represent total P, inorganic P, and organic P, respectively.

### Microbial biomass, PLFAs, and ALP activity analysis

Microbial biomass was measured according to the chloroform fumigation extraction method (Witt et al., 2000). We prepared two samples of the same weight of soil aggregate; one was extracted directly, while the other was fumigated at 25°C for 48 h. For microbial biomass carbon (MBC) and microbial biomass nitrogen (MBN), the samples were extracted with 0.5 M K_2_SO_4_ and measured using a total organic carbon analyzer (Vario TOC Cube, Elementar, Langenselbold, Germany). For microbial biomass P (MBP), the samples were extracted with 0.5 M NaHCO_3_ and measured using an automated discontinuous flow analyzer (Smartchem 450, AMS, Italy). The values of MBC, MBN, and MBP were calculated by subtracting the fumigated sample from the unfumigated sample based on a conversion coefficient of 0.45, 0.54, and 0.40, respectively (Ju et al., 2023). The value obtained from the unfumigated sample, using a conversion coefficient of 0.45, was used to represent dissolved organic carbon (DOC).

We further analyzed the microbial community using the phospholipid fatty acid (PLFA) profiling method (Bossio and Dcow, 1998). Briefly, 4.00 g freeze-dried soil aggregate was extracted with 5 mL of phosphate buffer, 6 mL of chloroform, and 12 mL of methanol. The extracted lipids were then separated using an extraction column methylated with 0.2 M methanolic KOH. This purified sample was measured with a gas chromatography–mass spectrometry (GC–MS) system (Agilent, CA, USA). The absorption peaks of 16:1ω5c, summed Feature 5, and 8:1ω9c were identified as fungi. The peaks of cy17:0, cy19:0, summed Features 8 and 3, a15:0, i15:0, i16:0, a17:0, i17:0, 10Me17:0, 10Me18:0, 5:00, 17:00, 18:1ω5, and 18:1ω7 were identified as bacteria (Olsson et al., 1995; Wang et al., 2023).

We measured ALP activity using a fluorometric assay method (DeForest, 2009). Briefly, 1.00 g of a fresh soil aggregate sample was homogenized with an acetate buffer to create a soil suspension. Then, the acetate buffer, soil suspension, standard solution, and substrate solution were added sequentially to a black 96-well microplate. The microplate was incubated at 25°C for 3 h. After stopping the reaction with a sodium hydroxide solution, we measured the fluorescence intensity using a microplate reader (ND-1000, Turner Designs, Wilmington, DE, USA). Each soil aggregate sample was measured six times, and the average value was used to calculate the enzyme activity (Shi et al., 2018).

### Statistical analyses

Before statistical analysis, all data were examined for normality and standardized if necessary. To assess the effects of four treatments on soil P fractions, plant, soil, and microbial properties, we conducted linear mixed-effects models. In these models, P fractions and explanatory variables served as fixed effects, while the block was treated as a random effect. Based on the fitted linear mixed-effects models, a one-way ANOVA with Tukey’s HSD test (Escobar-Bravo et al., 2022) was performed to compare soil P fractions across the four treatments within each soil aggregate.

To explore how these environmental explanatory variables impact soil P fraction dynamics, the Spearman correlation analysis was performed using the “psych” package to evaluate the relationships between soil P fractions and explanatory variables. A random forest analysis was conducted to rank the relative importance of explanatory variables in driving the soil P fractions. Moreover, the Mantel test (Wittes and Wallenstein, 1987) was performed to assess correlations among explanatory variables using the “linkET” and “dplyr” packages. We identified drivers that co-occur in both the random forest and Mantel analysis to build a causal analysis model. Piecewise structural equation modeling (SEMs) was developed to analyze the direct and indirect effects of these drivers on P fractions. All statistical analyses were conducted using R software v4.4.2.

## Results

### Effects of mowing and P addition on soil P fractions at soil aggregate levels

**Figure 1:**
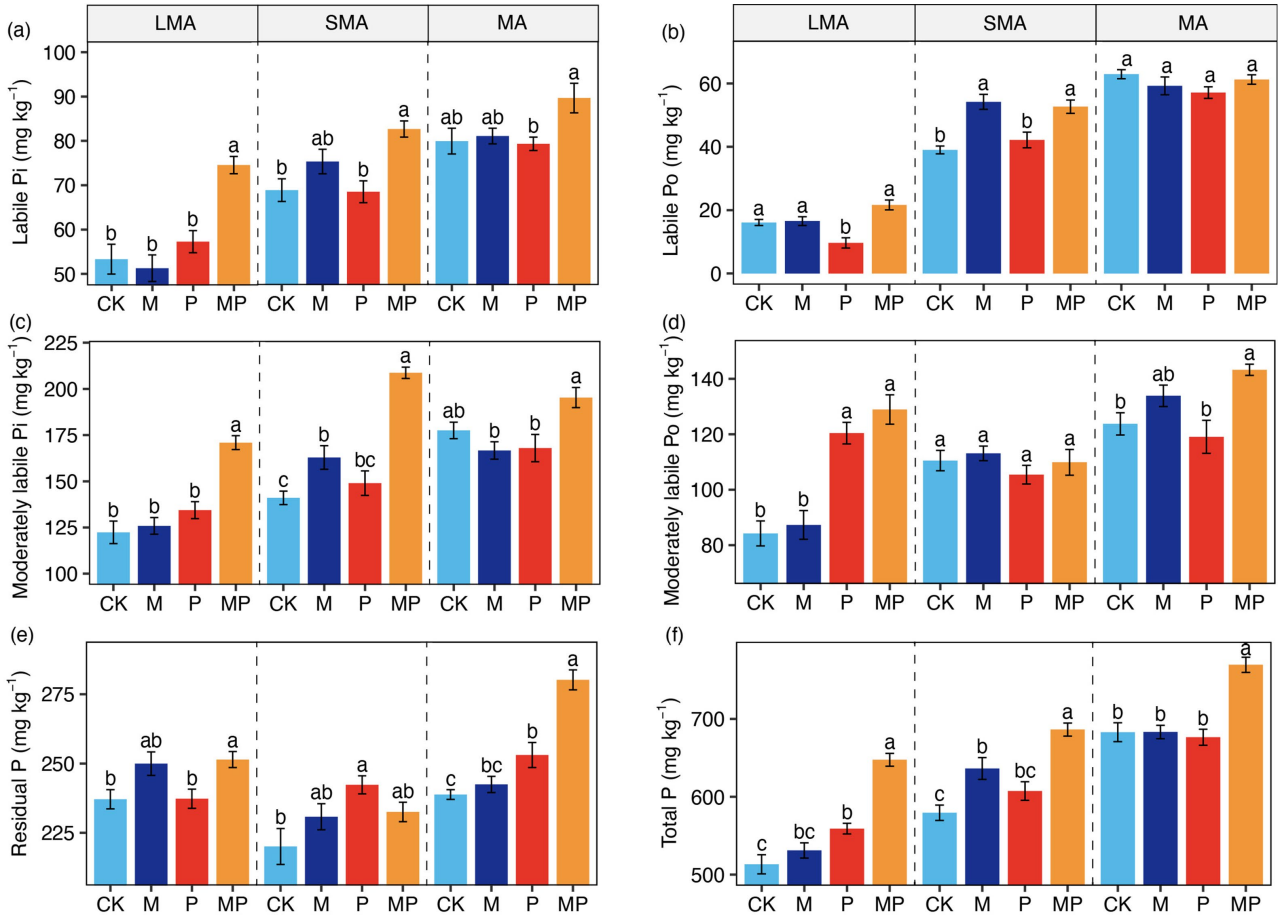
Phosphorus (P) fraction concentrations in large macroaggregates (LMA), small macroaggregates (SMA), and microaggregates (MA) under mowing and P addition. For each aggregate, different letters **(a–f)** denote significant differences in P fractions among the four treatments. CK, control; M, mowing; P, P addition; and MP, combined mowing and P addition. Values are means ± standard errors (n = 6).

The treatment had no significant effect on the proportion of soil aggregates (Supplementary Figure S2). The effects of mowing and P addition on soil P fractions varied with soil aggregate size. The interactive effect of mowing and P addition increased overall P levels across soil aggregates (Figure 1f). Additionally, this interactive effect enhanced labile Po in SMA (Figure 1b), as well as moderately labile Po and residual P in both LMA and MA (Figure 1d). In LMA, P addition raised moderately labile Po (Figure 1d). In SMA, mowing increased labile Po and moderately labile Pi (Figures 1b,c). P addition also increased residual P in both SMA and MA (Figure 1e).

### Effects of mowing and P addition on plant and soil abiotic properties

**Figure 2:**
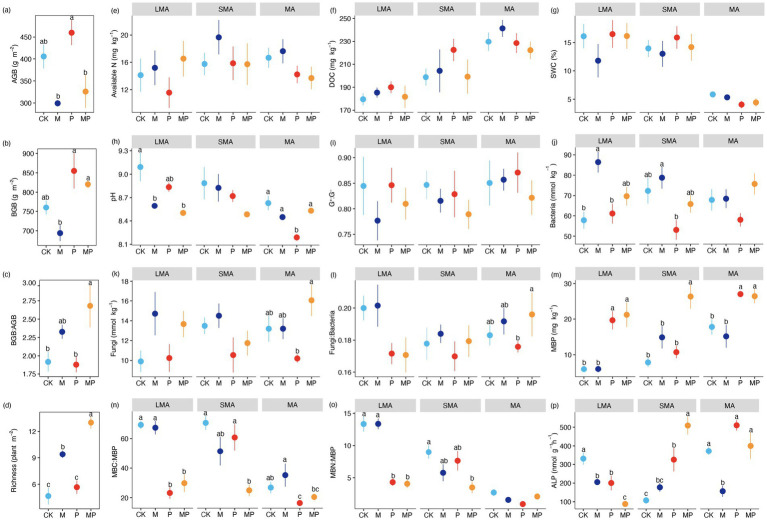
Environmental variables affected by mowing and P addition in LMA, SMA, and MA. For each aggregate, different letters **(a–p)** denote significant differences in P fractions among the four treatments. Values are means ± standard errors (n = 6). See Figure 3 for the meaning of acronyms.

The interactive effect of mowing and P addition increased BGB: AGB (Figure 2b) and plant richness (Figure 2d). Mowing decreased plant biomass (AGB and BGB) (Figures 2a,b) while increasing the BGB: AGB ratio (Figure 2c) and plant richness (Figure 2d). P addition increased both AGB and BGB (Figures 2a,b). Variations in soil abiotic properties were observed across different soil aggregate size classes. Soil DOC concentration and pH decreased with increasing soil aggregate size (Figures 2f,h), whereas SWC decreased as aggregate size increased (Figure 2g). In large macroaggregates, the interactive effect of mowing and P addition, as well as mowing alone, decreased soil pH (Figure 2h). P addition lowered pH in MA (Figure 2h). Conversely, available N, DOC, and SWC showed no statistically significant differences among the four treatments across aggregate sizes (Figures 2e–g).

### Effects of mowing and P addition on soil microbial properties

The effects of mowing and P addition on soil microbial communities depended on aggregate size classes. Soil MBP and ALP were higher in MA than in LMA (Figures 2m,p), whereas the microbial biomass carbon and microbial biomass phosphorus (MBC: MBP) and MBN: MBP ratios displayed contrasting trends (Figures 2n,o). In LMA, the interactive effect of mowing and P addition significantly increased MBP (Figure 2m) while decreasing MBC: MBP, MBN: MBP, and ALP (Figures 2n–p). Conversely, mowing increased MBC: MBP, MBN: MBP, and bacterial biomass (Figures 2n,o,j), but reduced ALP activity (Figure 2p). Additionally, P addition also decreased ALP but increased MBP (Figures 2m,p). In SMA, the interactive effect of mowing and P addition increased MBP and ALP while decreasing MBC: MBP and MBN: MBP (Figures 2m–p). Notably, P addition alone further enhanced ALP activity but reduced bacterial biomass (Figures 2p,j). In MA, the interactive effect of mowing and P addition increased MBP (Figure 2m), while mowing reduced ALP (Figure 2p). P addition enhanced MBP (Figure 2m) but lowered the MBC: MBP (Figure 2n).

### Response of soil P fractions and factors driving under mowing and P addition

**Figure 3:**
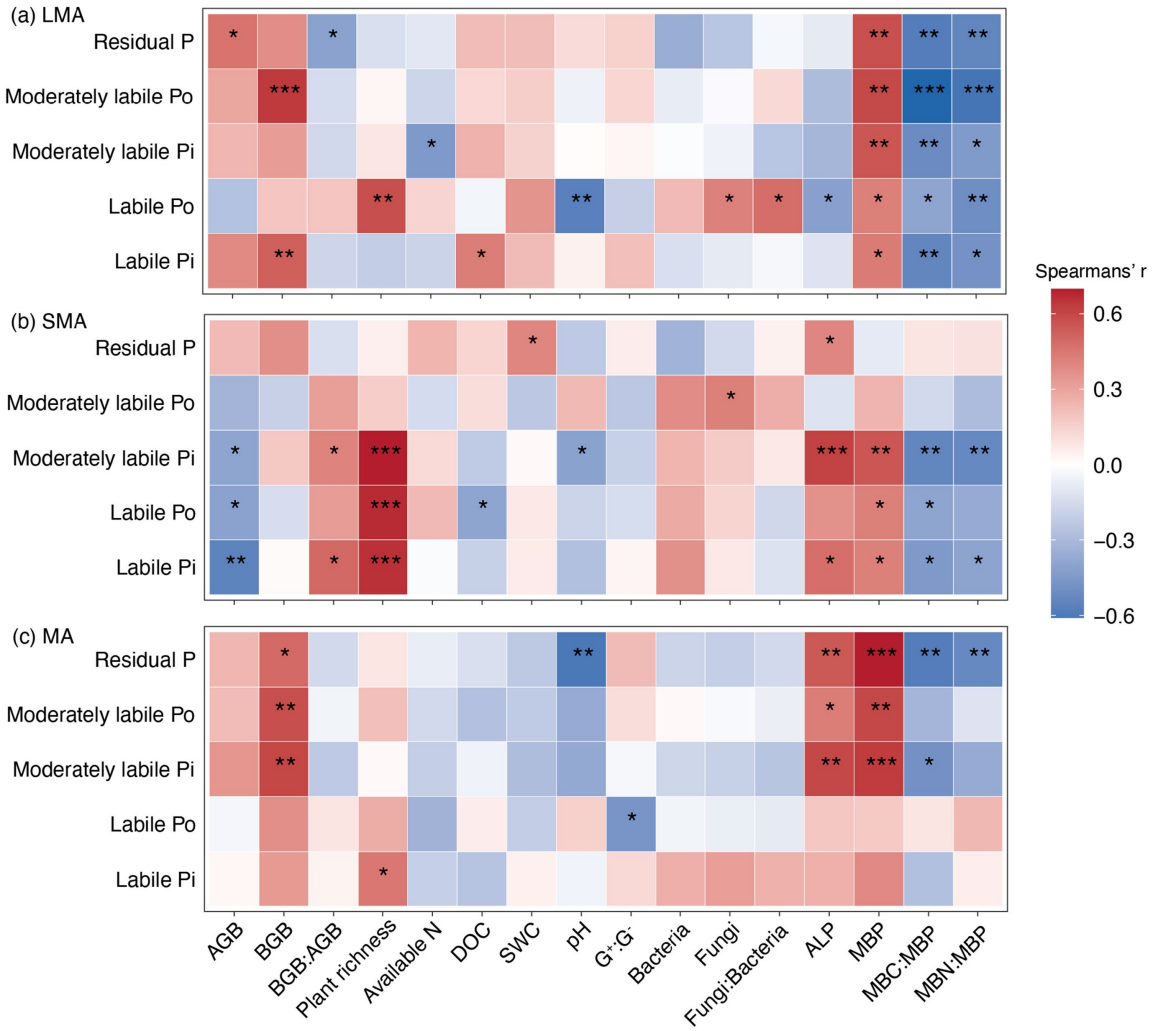
Relationships between P fractions and environmental variables in **(a)** LMA, **(b)** SMA, and **(c)** MA. AGB, plant aboveground biomass; BGB, plant belowground biomass; available N, available nitrogen; DOC, dissolved organic carbon; SWC, soil water content; G^+^: G^−^, Gram-positive and Gram-negative bacteria biomass ratio; Bacteria, bacterial PLFAs; Fungi, fungi PLFAs; fungi:bacteria, the ratio of fungi to bacterial PLFAs; ALP, alkaline phosphatase; MBP, microbial biomass phosphorus; MBC: MBP, the ratio of microbial biomass carbon to microbial biomass phosphorus; MBN: MBC, the ratio of microbial biomass nitrogen to microbial biomass phosphorus. The significant effects are marked with asterisks. *, **, and *** represent significant levels at *p <* 0.05, *p <* 0.01, and *p <* 0.001, respectively.

Soil P fractions exhibited distinct correlation patterns with biotic and abiotic drivers across soil aggregates (Figure 3). In LMA, all soil P fractions were significantly positively correlated with MBP but negatively correlated with the microbial stoichiometric ratio (i.e., MBC: MBP and MBN: MBP, Figure 3a). In SMA, the labile Pi, labile Po, and moderately labile Pi were strongly positively correlated with ALP and MAP, whereas they showed negative correlations with the microbial stoichiometric ratio. Conversely, in MA, moderately labile Pi, moderately labile Po, and residual P were strongly positively correlated with ALP and MAP, while they were negatively correlated with the MBC: MBP and MBN: MBP (Figure 3c).

**Figure 4:**
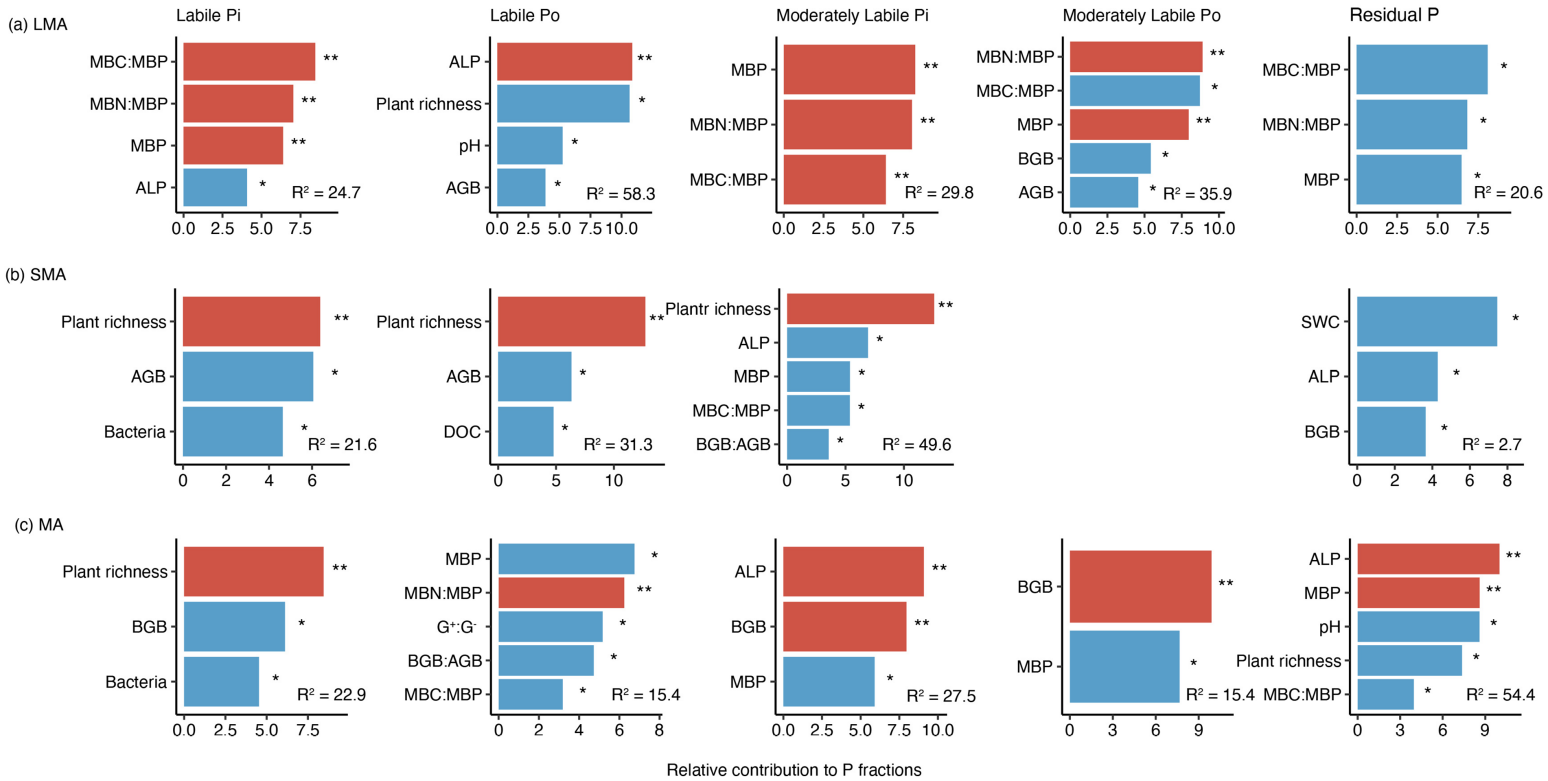
Ranking of environmental variables based on their contribution to P fractions in **(a)** LMA, **(b)** SMA, and **(c)** MA. The significant effects are marked by asterisks. * and ** represent significant levels at *p <* 0.05 and *p <* 0.01, respectively. See Figure 3 for the meaning of acronyms. Only significant variables are displayed. Contributions of other variables are shown in Supplementary Figure S3.

**Figure 5:**
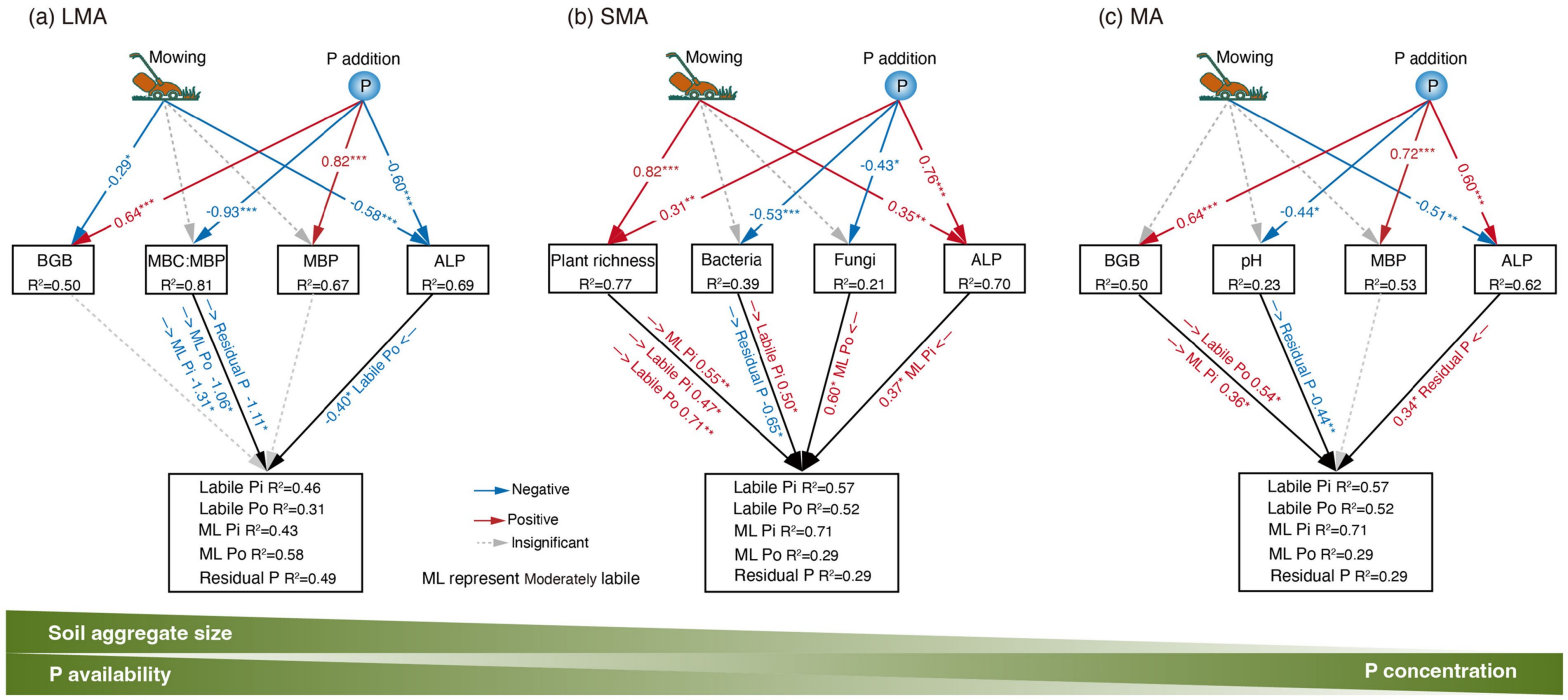
SEM results showing the effects of drivers on P fractions’ dynamics in **(a)** LMA model: goodness of fit (Fisher’s C, *p-*values) for Labile Pi: 10.93, 0.535; Labile Po: 11.46, 0.49; Moderately Labile Pi: 10.66, 0.558; Moderately Labile Po: 13.48, 0.489; Residual P: 11.56, 0.481. **(b)** SMA model: goodness of fit (Fisher’s C, *p-*values) for Labile Pi: 10.73, 0.552; Labile Po: 8.53, 0.577; Moderately Labile Pi: 13.35, 0.205; Moderately Labile Po: 7.73, 0.72; Residual P: 9.04, 0.7. **(c)** MA model: goodness of fit (Fisher’s C, *p-*values) for Labile Pi: 4.78, 0.965; Labile Po: 13.93, 0.604; Moderately Labile Pi: 8.19, 0.879; Moderately Labile Po: 7.60, 0.909; Residual P: 6.84, 0.941. The significant pathway is shown, with blue and red representing negative and positive effects, respectively. *, **, and *** indicate significant levels at *p <* 0.1, *p <* 0.05, and *p <* 0.001, respectively. See Figure 3 for the meanings of acronyms.

In LMA, the increase in moderately labile Pi and moderately labile Po resulted from the decreased levels of microbial biomass carbon and microbial biomass phosphorus (MBC: MBP), which were stimulated by P addition (Figures 3a, 4a, 5a; Supplementary Figure S1a). In SMA, P fractions were primarily influenced by plant richness, bacterial and fungal abundance, and ALP (Figures 3b, 4b). P addition and mowing increased plant richness, which in turn increased labile Pi and moderately labile Pi (Figure 5b). Meanwhile, P addition increased residual P by decreasing bacterial abundance (Figure 5b). Conversely, in MA, P addition increased residual P by increasing ALP (Figure 5c). The available P (the sum of labile Pi and labile Po) increased by 74.13, 55.01, and 9.50% in large macroaggregates (LMA), small macroaggregates (SMA), and microaggregates (MA), respectively.

## Discussion

Our study demonstrated that mowing and P addition significantly increased all P fractions and total P in LMA (Figure 1), primarily due to changes in soil microbial biomass and its stoichiometric ratios (Figures 2–5; Supplementary Figure S1a). Phosphorus (P) addition affects P availability and microbial P mobilization by altering soil P fractions (Chen et al., 2022). Furthermore, P addition can contribute to P storage in microbial biomass by increasing soil P concentrations (Jiang et al., 2013; Spohn and Widdig, 2017; Chen and Xiao, 2023). Our results showed that P addition and its interaction with mowing significantly increased MBP in LMA (Figure 2m). Our findings are consistent with previous research, as evidenced by a meta-analysis examining the effects of nutrient addition on soil microorganisms, which reported that P addition significantly increases MBP (Wu et al., 2022). P stored within microorganisms ultimately restores soil available P through microbial transformation (Kwabiah et al., 2003). Our results showed a significant positive correlation between MBP and all P fractions (Figure 3a), indicating that variations in MBP had a substantial impact on the levels of P fractions.

The results showed that the reduction in MBC: MBP significantly increased moderately labile Pi, moderately labile Po, and residual P (Figure 5a), ultimately leading to a 74.13% increase in available P. Carbon sources are essential for soil microbial P transformation and microbial utilization of soil P (Jiang et al., 2013). Studies on the effects of carbon inputs on soil P fractions and microbial biomass found that carbon nutrition significantly increased soil P availability by stimulating microbial activity (Wang et al., 2024). Analysis revealed that both mowing and P addition tended to increase soil DOC, and the interaction between P addition and mowing significantly reduced MBC: MBP (Supplementary Figures S21f,n). This suggests that microorganisms have a high P use efficiency, but this improvement comes at the cost of greater carbon and energy loss. These results indicate that microorganisms exhibit high P use efficiency, but this improvement entails increased carbon and energy loss. The present study aligns with Huang et al. (2021), who demonstrated that increased soil carbon significantly reduced MBC: MBP and stimulated microbial carbon metabolism. This, in turn, increased the carbon demand, which might play a key role in regulating microbial involvement in soil P transformation. This suggests that microbial demand for carbon may contribute to soil P dynamics (Heuck et al., 2015; Chen et al., 2020).

In SMA, plant richness, bacteria abundance, fungi abundance, and ALP are the primary drivers of variation in soil labile Pi, labile Po, and moderately labile Pi levels (Figures 2–5; Supplementary Figure S1b). It was observed that plant richness was primarily associated with the concentrations of labile Pi, labile Po, and moderately labile Pi (Figure 5b). Both mowing alone and the combination of mowing and P addition significantly increased plant richness (Figure 2d). Previous research on P cycling in grassland ecosystems has demonstrated that more diverse plant communities can utilize soil P more efficiently than less diverse communities, suggesting that higher plant species diversity enhances soil P cycling (Oelmann et al., 2021). The total P availability is directly linked to plant species richness (Chen et al., 2022). This also provides evidence that facilitative effects between plant communities and resource allocation enhance plant P uptake and turnover, thereby increasing soil P availability (Karanika et al., 2007; Tang et al., 2021).

In addition, under diverse plant mixtures, soil carbon stocks increase, which may result from the enhanced allocation of photosynthetic products to underground parts driven by mowing treatment (Zhai et al., 2023). Given that soil carbon serves as an energy source for heterotrophic soil microbial communities, increased plant species richness may stimulate microbial growth by enhancing soil carbon input (Bastida et al., 2021). A global meta-analysis of plant richness and microbial community structure reported that microbial biomass, bacterial abundance, fungal abundance, and the ratio of fungi to bacteria all increased under conditions of higher plant richness (Chen et al., 2019). The present results show that long-term mowing significantly increased plant richness and tended to increase soil fungal abundance, bacterial abundance, and the fungi:bacteria ratio (Figures 2d,j–l). In addition to this observation, the increase in soil microbial abundance may stimulate extracellular enzymes associated with soil P dynamics, thereby affecting microbial nutrient use efficiency (Gilmullina et al., 2020). The current results show that long-term P addition and the interaction between mowing and P addition significantly increased ALP (Figure 2p). The SMA may exhibit higher microbial nutrient use efficiency (Li et al., 2023). It was emphasized that mowing and P addition increase available P primarily by enhancing plant richness and ALP activity in SMA.

The results indicate that MA had the highest total P concentration under the same treatment (Figure 1). BGB, pH, MBP, and ALP were the explanatory variables most strongly associated with variation in moderately labile Pi, moderately labile Po, and residual P (Figures 2–5; Supplementary Figure S1c). Our results showed that P addition significantly increased residual P content by enhancing ALP activity (Figure 5c). The higher levels of total P concentration in MA may result from available P ions forming the highest percentage of residual P through reactions with minerals (Wright, 2009). Our study showed that P addition significantly increased residual P (Figure 1e). Additionally, soil MA provides better protection for soil nutrients due to limited water and oxygen diffusion (Fonte et al., 2014), which may limit the capacity of microorganisms to mobilize soil nutrients. Although previous studies have shown that water and oxygen diffusion rates are limited (Six et al., 2004), ALP activity involved in soil P cycling is still higher in MA than in LMA and SMA (Figure 2p). In grassland ecosystems, P addition significantly enhanced ALP activity (Zhang et al., 2024).

We emphasize that in MA, enhanced ALP and BGB growth induced by mowing and P addition are the primary drivers of improved soil P fractions and, thus, P availability (Figures 3c, 5c). Long-term P addition significantly increased BGB (Figure 2h). In addition, the interaction of mowing and P addition increased the BGB: AGB ratio (Figure 2c), suggesting that their interaction stimulates root growth and may influence soil nutrient cycling through nutrient uptake and root secretions (Lange et al., 2015). The results indicate that a significant increase in BGB enhances labile Po and moderately labile Pi (Figure 5c). Low molecular weight organic acids in root secretions (citric acid and malic acid) contain at least one carboxyl group (COOH or COO^−^) (Wang and Lambers, 2020). These organic anions increase available P in the soil solution by competing with P cations for adsorption sites. Increased H^+^ in the soil solution can inhibit calcium P precipitation through cation exchange, which in turn increases P availability (Penn and Camberato, 2019). Our results showed the lowest soil pH values in MA and a negative correlation with residual P (Figure 3c), suggesting that increased soil solution H^+^ may interact with residual P to increase soil P availability. Decreased soil pH accelerates the conversion between soil P fractions and increases soil available P (Menezes-Blackburn et al., 2018; Wang et al., 2022).

## Conclusion

This study highlights differences in microbial drivers regulating soil P fractions in response to mowing and P addition across different soil aggregate sizes. The increase in soil P fractions in LMA may result from P addition enhancing MBP, which is subsequently mobilized and activated in response to increases in soil carbon stimulated by mowing, thereby improving soil P fractions. Increased plant richness in SMA enhances soil carbon input and increases ALP activity secreted by soil microorganisms, thereby increasing P fractions, except for moderately labile Pi. The highest soil P fraction levels in MA may be due to low water content restricting microbial mobility and P mobilization. Consequently, MA retains high P fractions but exhibits a lower rate of available P accumulation. Overall, our results provide a microbially based mechanistic understanding of soil P dynamics and availability driven by grassland management and nutrient addition. Differences in the concentration and distribution of soil P fractions across aggregate sizes underscore the importance of soil aggregates in regulating grassland soil P dynamics. These findings provide critical insights to investigate the role of soil aggregates in regulating microbially driven soil P cycling, thereby supporting the development of sustainable grassland management strategies. It was noted that microbial biomass, enzyme activity, and PLFA profiles were used as proxies for microbial properties in this study. Future research integrating high-throughput sequencing and functional gene analysis is necessary to complement the findings and more precisely reveal the microbial mechanisms of P cycling.

## Data Availability

The raw data supporting the conclusions of this article will be made available by the authors, without undue reservation.

## Footnotes

^1^ https://data.cma.cn/site/index.html
