# Supplementary material for: Soil aggregates regulate microbial drivers of phosphorus fractions under mowing and phosphorus addition
Source: Front Microbiol. 2025 Oct 30;16:1671636. doi: 10.3389/fmicb.2025.1671636 (PMC12611934; doi:10.3389/fmicb.2025.1671636)
Supplement: Supplementary file 1 [file Data_Sheet_1.docx]

**Soil aggregates regulate microbial drivers of phosphorus fractions under mowing and phosphorus addition**

Xiuping Li, Haiying Cui**^*^**^,^
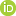
, Shanling Wang, Baoshuang Hu, Huiliang Zhai, Jiaxin Hu, Xia Peng, Muhammad Shakir, Wei Sun^,^**^**^**
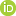


Institute of Grassland Science, School of Life Science, Northeast Normal University, Key Laboratory of Vegetation Ecology, Ministry of Education, Jilin Songnen Grassland Ecosystem National Observation and Research Station, Changchun, 130024, China

**Corresponding author:**

* Haiying Cui, E-mail: [cuihy608@nenu.edu.cn](mailto:sunwei@nenu.edu.cn)

**Wei Sun; E-mail: [sunwei@nenu.edu.cn](mailto:sunwei@nenu.edu.cn)

**ORCID ID**

Haiying Cui
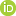
 <https://orcid.org/0000-0003-4993-2231>

Wei Sun
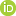
 <https://orcid.org/0000-0002-1601-2159>


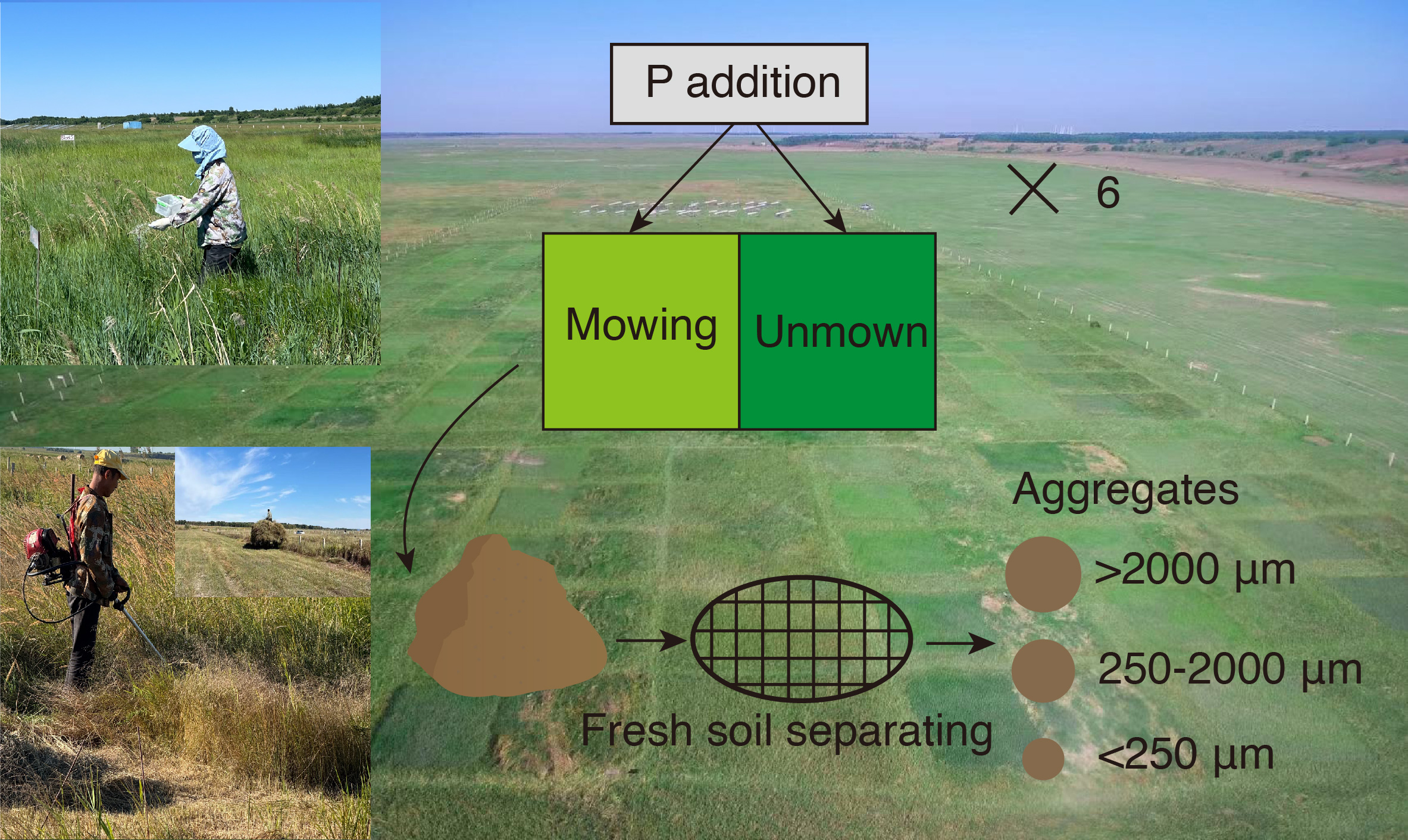


**Fig. S1.** Field with mowing and P addition, and schematic flow of the aggregate dry sieving method。


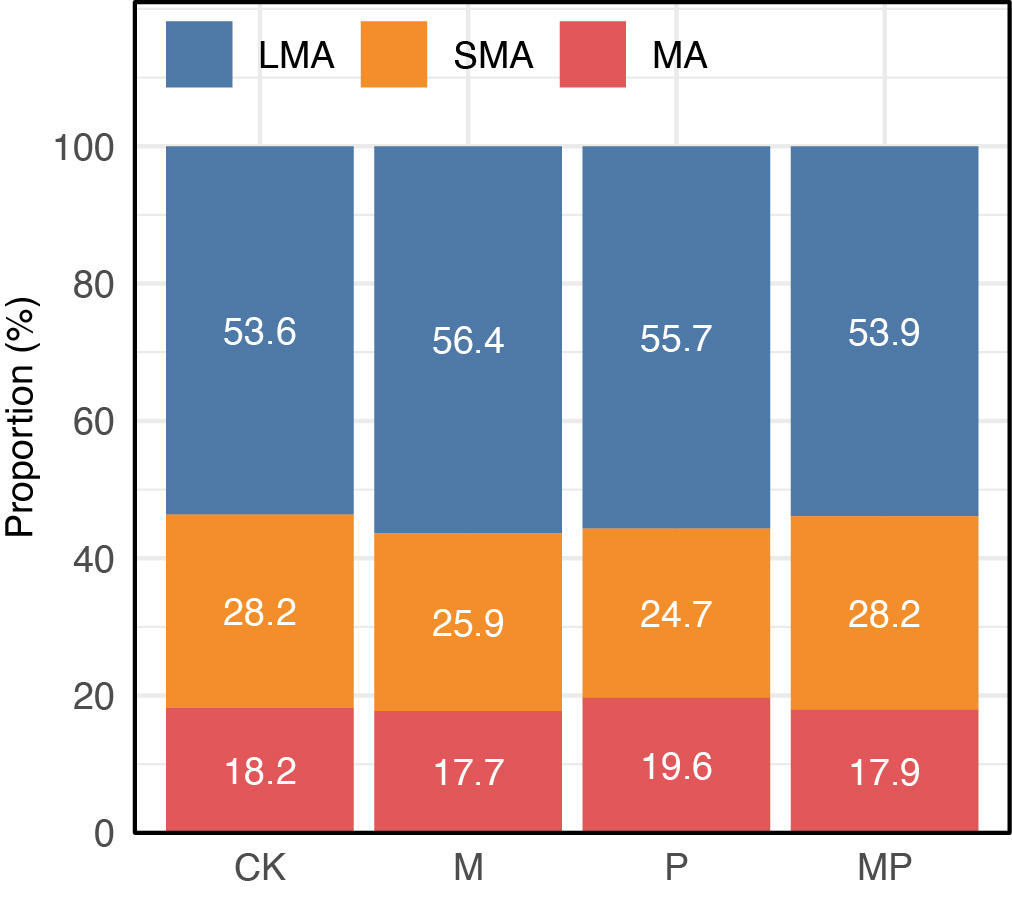


**Fig. S2.** soil aggregate proportion under mowing and phosphorus addition.


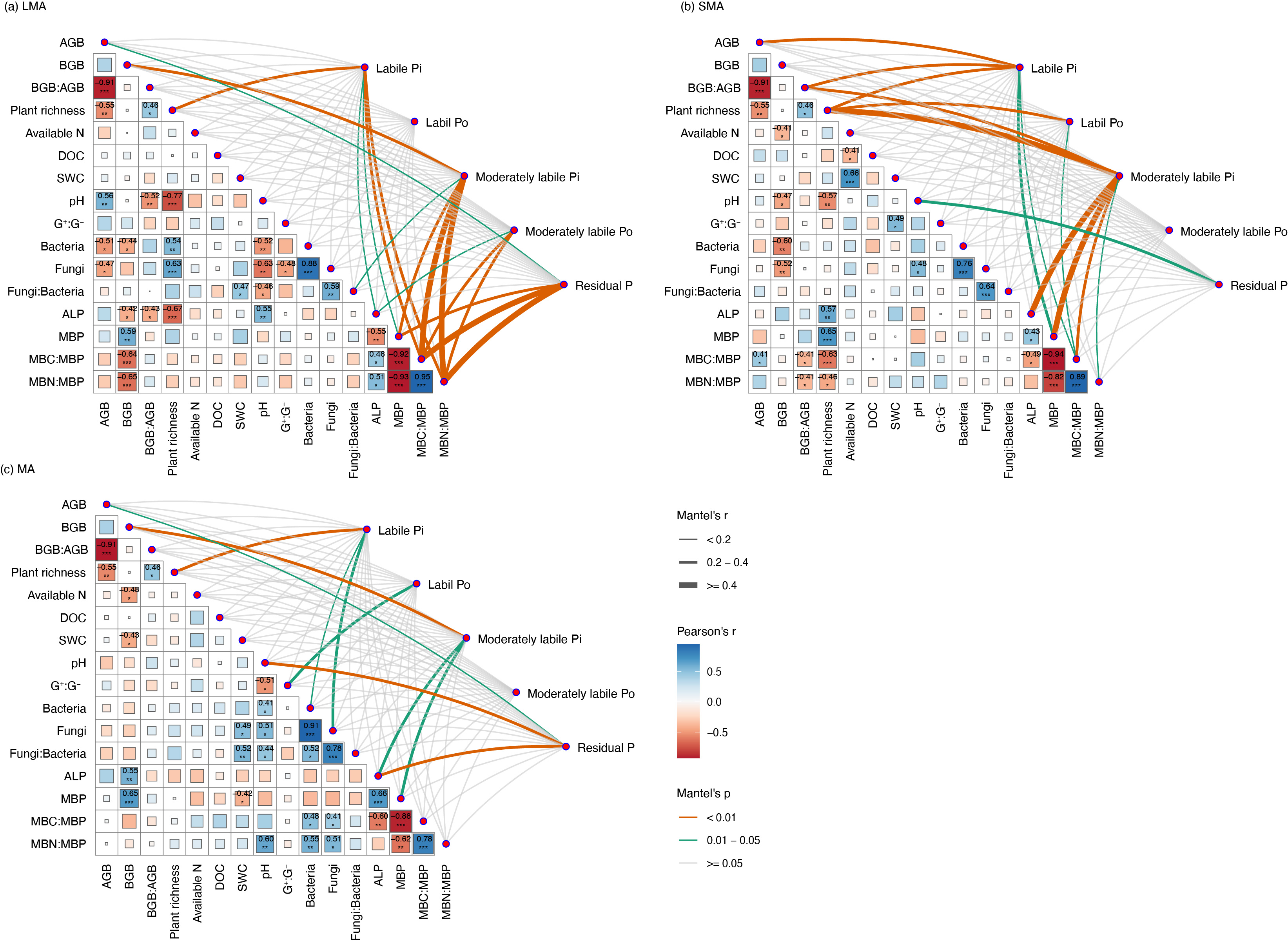


**Fig. S1.** Mantel test showing the relationships between environmental variable factors and the strength of correlation between P fractions and environmental variables in LMA (a), SMA (b), and MA (c). The significantly effect are marked by asterisks. *, ** and *** represent significant levels at p < 0.05, p < 0.01 and p < 0.001, respectively. See Fig. 2 for meaning of acronyms.


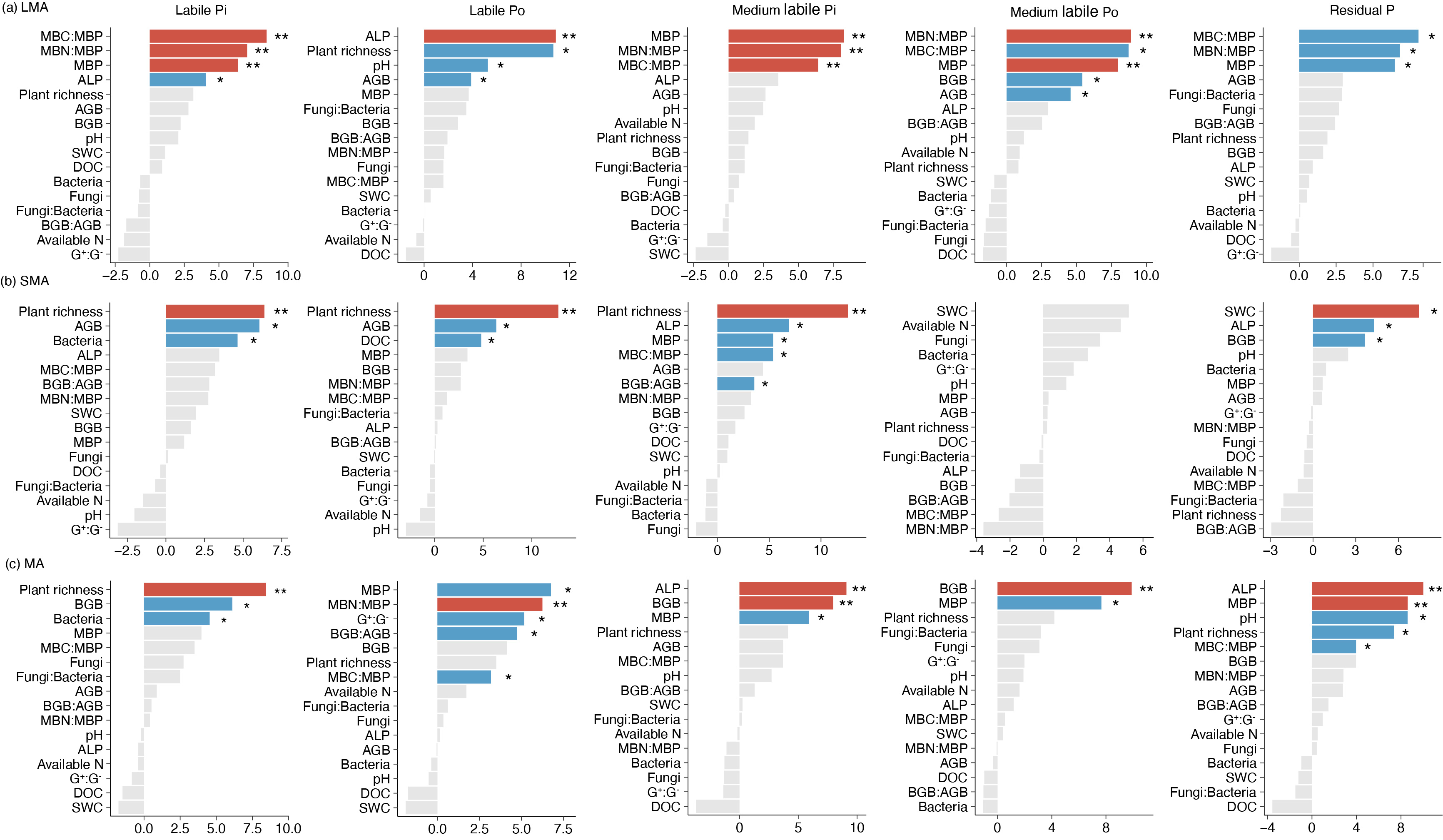


**Fig. S2.** Ranking of environmental variables based on their contribution to P fractions in (a) LMA, (b) SMA, and (c) MA. The significantly effect are marked by asterisks. *, ** and *** represent significant levels at p < 0.05, p < 0.01 and p < 0.001, respectively. See Fig. 2 for meaning of acronyms. See Fig. 3 for model parameters.
